# The year in cardiovascular medicine 2021: imaging

**DOI:** 10.1093/eurheartj/ehac033

**Published:** 2022-03-08

**Authors:** Chiara Bucciarelli-Ducci, Nina Ajmone-Marsan, Marcelo Di Carli, Edward Nicol

**Affiliations:** 1 Royal Brompton and Harefield Hospitals, Guys' and St Thomas NHS Trust and School of Biomedical Engineering and Imaging Sciences, Faculty of Life Sciences and Medicine, King's College London, London, UK; 2 Department of Cardiology, Leiden University Medical Center, 2333 ZA Leiden, The Netherlands; 3 Cardiovascular Imaging Program, Department of Radiology, Heart and Vascular Center, Harvard Medical School, Boston, MA, USA; 4 Cardiovascular Imaging Program, Department of Medicine, Heart and Vascular Center, Harvard Medical School, Boston, MA, USA; 5 Division of Cardiovascular Medicine, Department of Medicine, Harvard Medical School, Boston, MA, USA; 6 Division of Nuclear Medicine and Molecular Imaging, Department of Radiology, Brigham and Women’s Hospital, Harvard Medical School, Boston, MA, USA

**Keywords:** Imaging, Echocardiography, CMR, Magnetic resonance, Cardiac CT, PET, SPECT, Ischaemic heart disease, Valvular heart disease, Amyloidosis

## Abstract

This article reviews the most relevant literature published in 2021 on the role of cardiovascular imaging in cardiovascular medicine. Coronavirus disease 2019 (COVID-19) continued to impact the healthcare landscape, resulting in reduced access to hospital-based cardiovascular care including reduced routine diagnostic cardiovascular testing. However, imaging has also facilitated the understanding of the presence and extent of myocardial damage caused by the coronavirus infection. What has dominated the imaging literature beyond the pandemic are novel data on valvular heart disease, the increasing use of artificial intelligence (AI) applied to imaging, and the use of advanced imaging modalities in both ischaemic heart disease and cardiac amyloidosis.

## Graphical Abstract

**Graphical Abstract:**
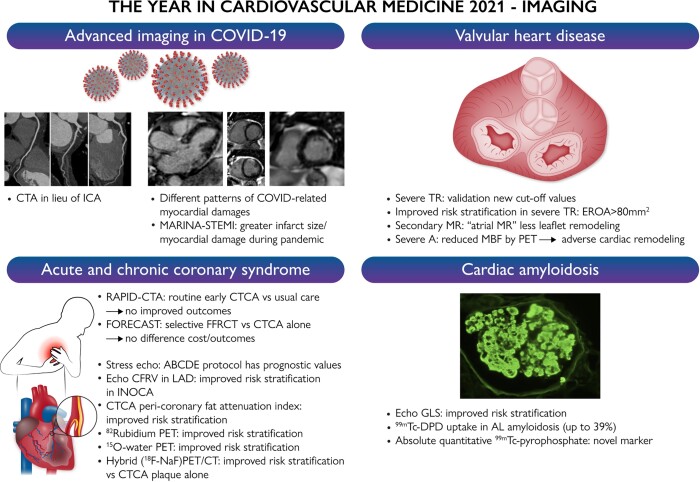
Summary of the most significant advances in cardiovascular imaging applied to acute and chronic coronary syndromes, valvular heart disease, cardiac amyloidosis, and myocardial damages caused by COVID-19 or the pandemic itself. CTCA, computed tomography coronary angiography; CMR, cardiac magnetic resonance; CFRV, coronary flow reserve velocity; PET, position emission tomography; TR, tricuspid regurgitation; MR, mitral regurgitation; AS, aortic stenosis; MBF, myocardial blood flow; GLS, global longitudinal strain. Images in part reproduced from Kotecha *et al*.^4^ and in part used from the creative common database.

## Introduction

Cardiovascular imaging continues to play an important role in improving risk stratification and management in patients with cardiovascular disease. In 2021, coronavirus disease 2019 (COVID-19) continued to impact the healthcare landscape, resulting in reduced access to hospital-based cardiovascular care for acute presentations, including reduced routine diagnostic cardiovascular testing. However, imaging has also facilitated the understanding of the presence and extent of myocardial damage caused by the coronavirus infection. What has dominated the imaging literature beyond the pandemic are novel data on valvular heart disease, the increasing use of artificial intelligence (AI) applied to imaging, and the use of advanced imaging modalities in both ischaemic heart disease and cardiac amyloidosis (CA) (*Graphical Abstract*).

## Cardiac involvement in the COVID-19 infection

The effect of the COVID-19 pandemic on cardiovascular imaging has been profound.^1^ Cardiovascular computed tomography (CCT) has been used successfully as a non-invasive alternative for both morphologic assessment, such as for left atrial appendage imaging in lieu of transoesophageal echocardiography,^2^ or computed tomography coronary angiography (CTCA) in lieu of invasive coronary angiography (ICA), effectively screening patients in whom revascularization is immediately required versus those who can be safely deferred until the acute pressures on global healthcare systems have lessened.^3^

The topic of myocardial injury caused by the COVID-19 infection had a prominent presence in both the scientific literature and in the media, on occasion causing concerns among physicians and members of the public regarding the extent to which the COVID-19 could affect the heart including in patients with mild symptoms not requiring hospitalization. In a multicentre UK study of 140 patients hospitalized with the COVID-19 infection and concomitant troponin rise, Kotecha *et al*.^4^ characterized myocardial injury using cardiac magnetic resonance (CMR). They identified myocarditis-like myocardial injury in 26% of patients, myocardial ischaemia and infarction in 22% of patients, and dual pathology (ischaemic and non-ischaemic) in 6% of patients. Also, the large majority of patients (89%) has a normal left ventricular (LV) systolic function [ejection fraction (EF) 67 ± 11%], providing evidence that myocardial injury during the COVID-19 infection can be detected but it has limited extent and minimal functional consequence.

**Figure 1:**
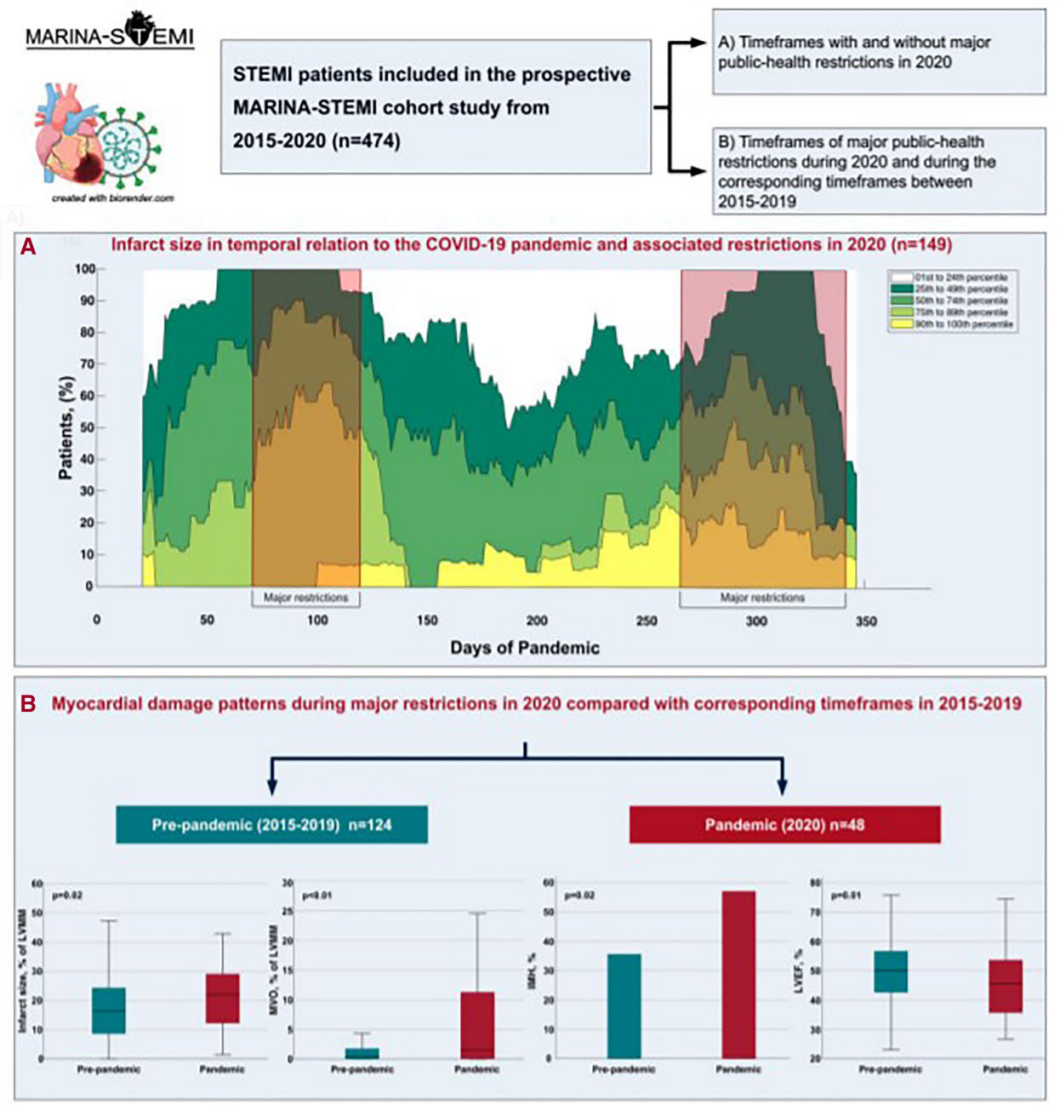
Infarct size and myocardial damage patterns in patients with ST-elevation myocardial infarction admitted during the COVID-19 pandemic. Reproduced with permission from Lechner *et al*.^5^

Results from the Magnetic Resonance Imaging in Acute ST-Elevation Myocardial Infarction (MARINA-STEMI) study explored the impact of the COVID-19 pandemic and associated public health restrictions on infarct severity and myocardial tissue damage. The study demonstrated a significant increase in myocardial damage assessed by CMR such as larger infarct size, more extensive microvascular obstruction, higher rate of intramyocardial haemorrhage, as well as a lower LVEF (*Figure 1*).^5^

## Acute coronary syndromes

The role of CTCA in the assessment of chest pain continues to evolve, and while the US guidelines and ESC guidelines support a greater role for CTCA in stable chest pain, recent data from the RAPID-CTCA study did not support the routine use of early CTCA in intermediate risk patients with acute chest pain and suspected acute coronary syndrome. Although it did reduce rates of ICA, it resulted in a modest increase in length of hospitalization, and did not alter overall coronary therapeutic interventions, or 1 year clinical outcomes.^6^

The role of adjunctive or advanced tools to improve clinical outcomes, resource allocation, and economic value of CTCA remained a significant focus of research in 2021. The recently published FORECAST Trial^7^ explicitly investigated whether selective FFR_CT_ vs. CTCA alone would improve economic and clinical care, but it showed no difference in either cost or clinical outcomes, despite a modest reduction in ICA.

## Chronic coronary syndromes

Stress echocardiography (SE) is an established technique to detect inducible myocardial ischaemia. A recent ABCDE protocol has been proposed which includes the analysis of the B lines by lung ultrasound (Step B), LV contractile reserve (Step C), coronary flow velocity reserve (CFVR) (Step D), and the heart rate reserve (Step E), in addition to the standard regional wall motion assessment. This extended protocol aims at capturing the various sources of vulnerability of patients with coronary artery disease (CAD), such as pulmonary congestion, myocardial impaired contractility, coronary microvascular dysfunction, and cardiac autonomic unbalance. Ciampi *et al*.^8^ demonstrated the prognostic value of the ABCDE-SE protocol in >3500 patients with chronic coronary syndromes, which were associated with an annual mortality rate ranging from 0.4% person-year for Score 0 up to 2.7% person-year for Score 5.

The iPOWER study^9^ tested the prognostic value of the echocardiographic assessment of CFVR in 1600 women with angina pectoris and no obstructive CAD. It demonstrates for the first time the independent association between CFVR and the composite endpoint of cardiovascular mortality, myocardial infarction, heart failure (HF), stroke, and coronary revascularization. This relatively simple and readily available technique could be therefore used in this patient population to diagnose coronary microvascular dysfunction, improve risk stratification, and identify patients eligible for intensified preventive treatment such as statins.

The role of coronary artery calcium scoring (CACS) as an independent risk factor for major adverse cardiac event (MACE) is well established, especially in patients aged >50 years; however, it is not without limitation, especially in those with a CACS of zero. Recent data from the SCOT-HEART group^10^ demonstrated 36% had a zero CACS, of which 2% had a obstructive disease, 2% had visually assessed adverse plaques, and 13% had a low-attenuation plaque burden (>4%). Myocardial infarction occurred in 41 patients, 10% of whom had a zero CACS.

The concept of reduced atherosclerotic risk with increased densification of calcium (especially following statin therapy) has also been a topic of debate in 2021 and is further supported in a multi-institutional cohort study of 857 patients who underwent serial CTCA, 2 or more years apart, with quantitative coronary plaques assessment throughout the entire coronary artery tree. The statin therapy was associated with volume decreases in low-attenuation plaque (*β*, −0.02; 95% CI, −0.03 to −0.01; *P* = 0.001), fibro-fatty plaque (*β*, −0.03; 95% CI, −0.04 to −0.02; *P* < 0.001), greater progression of high-density calcium plaque (*β*, 0.02; 95% CI, 0.01–0.03; *P* < 0.001), and 1K plaque (*β*, 0.02; 95% CI, 0.01–0.03; *P* < 0.001). When analyses were restricted to lesions without low-attenuation plaque or fibro-fatty plaque at baseline, the statin therapy was not associated with a change in overall calcified plaque volume (*β*, −0.03; 95% CI, −0.08 to 0.02; *P* = 0.24) but was associated with a transformation towards more dense calcium, which in turn was associated with less plaque progression.^11^

**Figure 2:**
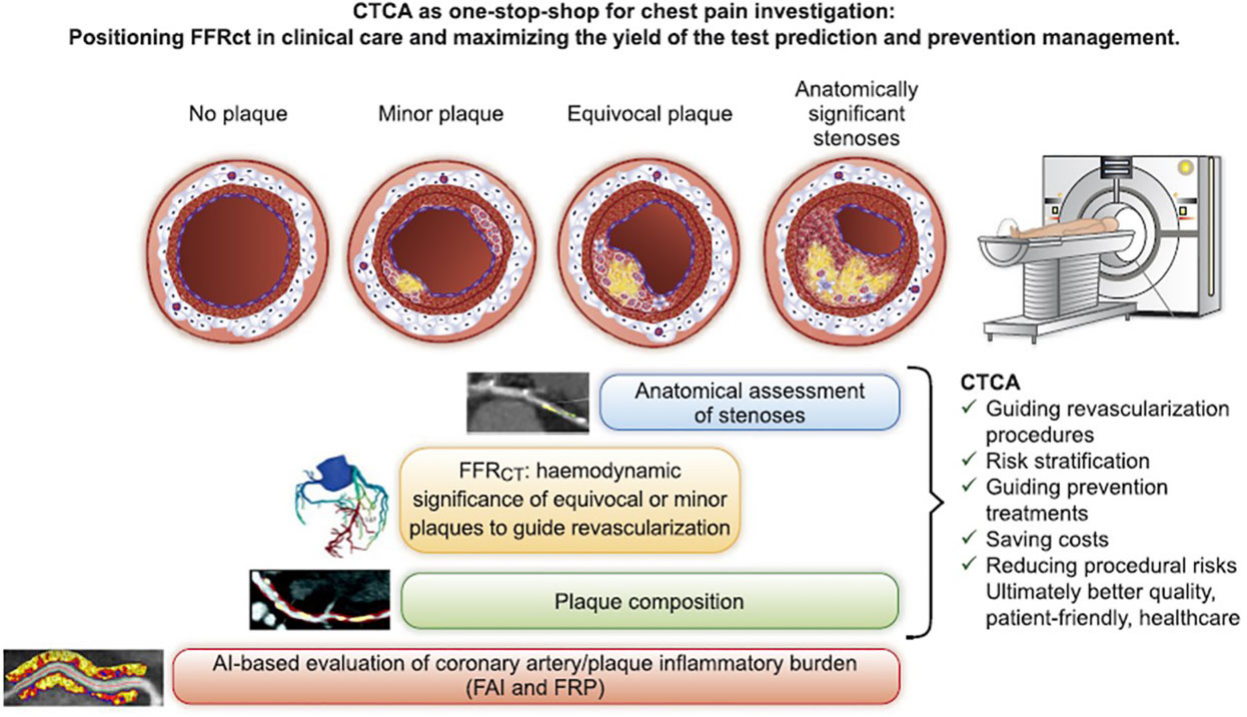
The advancing application of computed tomography coronary angiography imaging beyond stenosis assessment, to include plaque composition, computational flow dynamic, and machine learning algorithm-derived data, assessing visual and beyond the eye information to refine individual risk assessment. Reproduced with permission from Antoniades *et al*.^12^

Combining all the available data that can be accrued in a CTCA study in 2021 [calcium quantification, luminal assessment, plaque burden, morphology, and composition, as well as computational flow dynamics, and data from machine learning (ML) algorithms] is increasingly making CTCA an attractive, non-invasive imaging tool that is highly capable of identifying vulnerable patients who would most benefit from preventative therapies (*Figure 2*).^12^

Two new studies confirmed and expanded the role of quantitative position emission tomography (PET) for risk stratification in patients with suspected or known CAD. One large study including 5274 patients showed that the integration of stress myocardial blood flow (MBF) and flow reserve, termed coronary flow capacity (CFC), as quantified by ^82^Rubidium-PET improves risk stratification over conventional markers of risk (e.g. total perfusion deficit).^13^ Compared to patients with preserved CFC, those with the lowest CFC had the highest mortality risk (2.3 vs. 14%) and this risk was reduced with revascularization. A second study including 623 patients showed an association between the ischaemic burden derived by quantitative MBF data with ^15^O-water PET and MACE. They showed that patients with an ischaemic burden of at least 24% of the LV mass had worse outcomes than those with a lower ischaemic burden (annualized MACE: 2.8 vs. 0.6%). In multivariable modelling, a high ischaemic burden was prognostically incremental over pre-imaging variables.^14^ Two additional studies expanded the prognostic value of quantitative MBF with PET to patients with systemic inflammatory disorders without overt obstructive CAD, suggesting a prognostic association between systemic inflammation, diffuse atherosclerosis and coronary microvascular dysfunction.^15,16^

Kwiecinski *et al*.^17^ sought to leverage the power of molecular characterization of atherosclerotic plaques with hybrid CTCA and ^18^F-sodium fluoride (^18^F-NaF) PET/CT and prognostication using an AI-based survival model in patients with CAD. The study reports incremental value of quantitative ^18^F-NaF PET/CT over quantitative CT-based plaque analysis for the prediction of myocardial infarction (area under the receiver operating characteristic curve: 0.85 vs. 0.72, respectively).

Artificial intelligence continues to shape the applications of advanced cardiovascular imaging into more efficient and precise tools. Zhang *et al.*^18^ developed a CMR virtual native enhancement (VNE) imaging technology using AI to identify myocardial fibrosis without the need of a contrast agent. This new method was validated in 1348 patients with hypertrophic cardiomyopathy undergoing both late gadolinium enhancement (LGE) imaging post-contrast administration and VNE imaging. There was a good correlation between the two methods and the VNE had significantly better image quality than the LGE. Importantly, as the VNE images can be produced in <1 s, using this technology can significantly shorten a routine CMR protocol of cine and tissue characterization to 15 min.

Meanwhile, ML algorithms such as applied in the peri-coronary fat attenuation index (FAI) have shown significant promise in identifying those at risk of cardiovascular events, and when used as part of a cardiovascular risk assessment tool that integrates standardized FAI mapping together with clinical risk factors and plaque metrics, is now shown to provide robust individualized cardiovascular risk prediction.^19^

## Valvular heart disease

The grading of tricuspid regurgitation (TR) is currently based on 2D echocardiographic parameters, its cut-off values were never or poorly validated and they tend to capture only a limited range of regurgitation severity. The increasing awareness of the negative prognostic impact of TR and the introduction of new transcatheter valvular interventions, have raised the interest to improve TR grading. Muraru *et al*.^20^ assessed in almost 300 patients the relationship between TR severity and the composite endpoint of death and HF hospitalization in order to identify the threshold values of vena contracta width (VCW), effective regurgitant orifice area (EROA), regurgitant volume (RegVol), and regurgitant fraction (RegFr) to define low, intermediate, and high-risk TR. The obtained partition values (VCW > 6 mm, EROA > 0.30 cm^2^, RegVol > 30 mL, and RegFr > 45%) were lower than in current recommendations and may prevent the underestimation of TR severity, allowing patients to be treated earlier. Fortuni *et al*.^21^ studied 1129 patients with significant TR and found that although VCW < 7 mm performed well in stratifying the prognosis of patients with moderate vs. severe TR, EROA > 80 mm^2^ could better differentiate the risk of patients with more than severe TR. Based on these observations, VCW and EROA were combined into a novel algorithm to classify TR into three clinically meaningful categories: moderate, severe, and torrential, with the last one being independently associated with worse prognosis.

**Figure 3:**
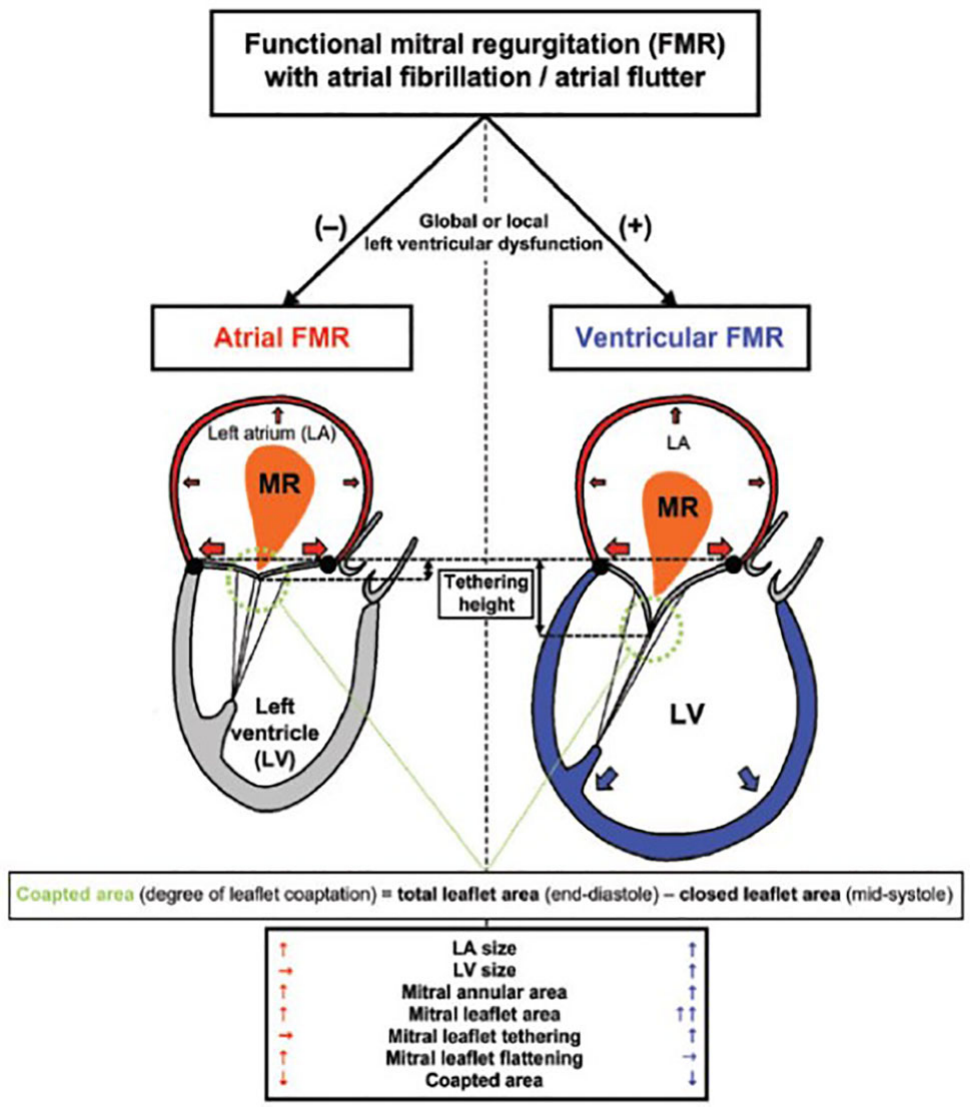
Geometrical differences in leaflet remodelling and tethering between atrial-functional mitral regurgitation and ventricular-functional mitral regurgitation in patients with atrial fibrillation: both leaflet remodelling and tethering are less in atrial-functional mitral regurgitation when compared with ventricular-functional mitral regurgitation with similar leaflet coaptation and regurgitation severity. Reproduced with permission from Uno *et al*.^22^

New important insights into mitral regurgitation (MR) have also been published this year. The concept of ‘atrial’ MR has been introduced for patients with secondary MR but normal LV dimension and function, typically observed in patients with atrial fibrillation or HF with preserved EF. Using three-dimensional transoesophageal echocardiography in 135 patients with secondary MR, Uno *et al.*^22^ showed that atrial MR (when compared with ventricular MR) is characterized by lower leaflet remodelling and less leaflet tethering (with ‘flattened’ annular shape) but with similar MR severity, suggesting the potential role of different therapeutic strategies for these two distinct secondary MR types (*Figure 3*). In primary MR, there is increasing evidence on arrhythmic mitral valve prolapse and its association with malignant ventricular arrythmias, particularly in the presence of mitral annular disjunction (MAD), as recently demonstrated in a large cohort (*n* = 595).^23^ MAD, as assessed by echocardiography, was independently associated with long-term increased arrhythmic events but without excess mortality.

As the interest in myocardial involvement in valvular heart disease continues to grow, Kwak *et al*.^24^ used ML in a large cohort of 440 patients with severe aortic stenosis from 13 centres to identify prognostically important CMR markers of myocardial remodelling and myocardial fibrosis in AS. They demonstrated that myocardial fibrosis (LGE percentage and extracellular volume of distribution) and biventricular remodelling (right ventricle EF and LV-indexed LV end-diastolic function) were the top predictors of survival in AS. The authors proposed combining these four imaging biomarkers into a score that could identify patients at high risk of mortality post-aortic valve replacement (post-AVR) and have the potential to optimizing the decision of AVR.

Zhou *et al*.^25^ investigated whether myocardial flow reserve (MFR) and stress MBF are associated with LV structure and function derangements, and whether these parameters improve after AVR. They demonstrated that in patients with aortic stenosis without obstructive CAD, MBF, and MFR were associated with adverse myocardial remodelling, including markers of myocardial injury and wall stress.

Finally, inflammation/infection imaging was used to diagnose infective endocarditis in pulmonary prosthetic valves. A multicentre registry of 66 children and adults with congenital heart disease reported that the use of ^18^F-FDG PET/CT was associated with moderately high sensitivity and specificity (79 and 73%, respectively) and a high positive predictive value (92%) but a low negative predictive value (47%). Importantly, a positive ^18^F-FDG PET/CT scan correctly reclassified 57% of possible to definite pulmonary prosthetic valve endocarditis.^26^

## Cardiac amyloidosis

Global longitudinal strain (GLS) by speckle tracking echocardiography has increasingly been validated as a sensitive tool of LV dysfunction in different clinical scenarios, showing an incremental diagnostic and prognostic value particularly in patients with preserved LVEF. As cardiac involvement is a major determinant of prognosis in amyloid light chain (AL), Cohen *et al*.^27^ demonstrated in the largest population to date (*n* = 615) of patients newly diagnosed with cardiac AL amyloidosis, baseline GLS was associated with mortality independently of standard biomarker-based criteria. Most importantly, only patients with a complete haematological response showed a significant improvement in GLS during follow-up, and an absolute improvement of GLS of 2% together with a cardiac biomarker response (decrease in proBNP) identified the subgroup of patients with the best survival. These data support the systematic measure of GLS in these patients in order to improve risk stratification and fine-tune therapy.

Two important studies expanding the role of nuclear molecular imaging in CA were published this year. One study addressed the question of the specificity of ^99m^Tc-SPECT imaging for the differentiation between transthyretin amyloidosis (ATTR) and AL amyloidosis.^28^ In a large cohort of AL patients, the study reported that ^99m^Tc-DPD uptake was present in 39% of patients but only 10% overall had Grade 2–3 cardiac uptake—threshold used to define the presence of ATTR amyloidosis. The data highlight the importance of ruling out AL amyloidosis in patients with ^99m^Tc uptake. The other study described the accuracy of absolute quantification of ^99m^Tc retention using advanced hardware.^29^ It found that all quantitative markers correlated with structural remodelling in CA. This approach opens an opportunity for early detection of CA, which is thought to be important to improve success of novel therapies.

## Cardiac masses

The use of CMR in assessing myocardial masses is well established, but there is a paucity of data on its diagnostic accuracy and prognostic role. In a multicentre study of 903 patients with cardiac masses undergoing CMR for further characterization, Shenoy *et al*.^30^ demonstrated how CMR identified no mass in 25%, pseudomass in 16%, thrombus in 16%, benign tumour in 17%, and malignant tumour in 23% of patients. Patients with CMR diagnoses of pseudomass and benign tumour had similar mortality to those with no mass, whereas those with malignant tumour [hazard ratio (HR) 3.31 (2.40–4.57)] and thrombus [HR 1.46 (1.00–2.11)] had greater mortality.

## Hypertension

**Figure 4:**
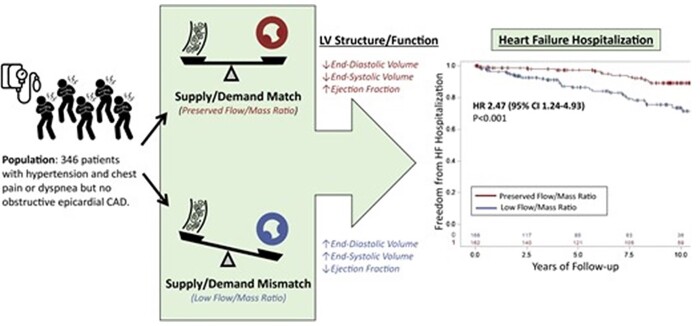
Symptomatic patients with hypertension are at increased risk of heart failure hospitalization when they have a low flow (myocardial perfusion)/mass ratio. Reproduced with permission from Brown *et al*.^31^

Brown *et al*.^31^ investigated the role of quantitative MBF with PET to understand maladaptive myocardial responses in hypertension, demonstrating that a lower stress myocardial flow indexed to myocardial mass (flow–mass ratio) to account for metabolic demand was independently associated with abnormal LV remodelling and with a higher risk of HF hospitalization and death in patients with hypertensive heart disease (*Figure 4*).

**Conflict of interest:** C.B.-D. is the chief executive officer (part-time) of the Society for Cardiovascular Magnetic Resonance; speaker's fees from Circle Cardiovascular Imaging, Bayer and Siemens Healthineers, she also participated in the medical advisory board of Bayer. N.A.-M. received speaker fees from GE Healthcare and Abbott Vascular; she also participated in the Medical Advisory Board of Philips Ultrasound. E.N. is the Secretary of the Society of Cardiovascular Computed Tomography; speaker fees from Siemens Healthineers, educational consultancy from GE, consultancy with Terarecon Inc, and advisory board of Caristo diagnostics. M.D.C. has none to declare.
